# Mild Traumatic Brain Injury Is Associated with Early Anterior Insula Hyperactivation During Appraisal of Fearful Stimuli in Trauma Survivors with Probable Post-Traumatic Stress Disorder

**DOI:** 10.3390/brainsci16090916

**Published:** 2026-08-28

**Authors:** Joyce Wu, Ahmed M. Afifi, Hong Xie, Lila E. Mahmoud, Sulaiman D. Aldoohan, Xin Wang, Cha-Hao Shih, Stephen R. Grider

**Affiliations:** 1 Department of Neurosciences and Psychiatry, University of Toledo, Toledo, OH 43614, USAhong.xie@utoledo.edu (H.X.); lila.mahmoud@rockets.utoledo.edu (L.E.M.); 2 Department of Emergency Medicine, University of Toledo, Toledo, OH 43614, USA; ahmed.abdelwahab@utoledo.edu (A.M.A.); chiahao.shih@gmail.com (C.-H.S.); stephen.grider@utoledo.edu (S.R.G.); 3 Department of Radiology, University of Toledo, Toledo, OH 43614, USA; sulaiman.aldoohan@utoledo.edu

**Keywords:** fMRI, mTBI, PTSD, motor vehicle collision, insular cortex, negative emotion

## Abstract

**Highlights:**

**What are the main findings?**

- Functional MRI findings suggest that previously reported left anterior insular cortex hyperactivation during the appraisal of fearful faces at three months after an MVC in survivors with probable PTSD was greater within two weeks after trauma among survivors who experienced mTBI and had probable PTSD at three months, compared to survivors who had probable PTSD without experiencing mTBI.
- Within-group analyses showed post-traumatic stress symptoms significantly decreased over time in MVC survivors without mTBI, but no significant decrease was observed in those with mTBI; however, overall time and time × group interactions were nonsignificant.

**What are the implications of the main findings?**

- Activation in the left anterior insular cortex to negative emotional stimuli may be greater in the early weeks post-trauma after MVC among probable PTSD survivors who experienced mTBI.
- Post-traumatic stress symptoms may lessen over the first three months after trauma in survivors who did not experience mTBI during MVC.

**Abstract:**

**Background**: Mild traumatic brain injury (mTBI) may rapidly alter neurocognitive function and has been associated with an increased risk for post-traumatic stress disorder (PTSD). However, neuroimaging investigations during the acute post-trauma phase remain sparse. We previously reported greater left anterior insular cortex (aIC) activation during the cognitive appraisal of fearful versus neutral emotional faces in survivors who did and did not exhibit probable PTSD at 3 months, but not at 2 weeks, after a motor vehicle collision (MVC). Given previous studies suggesting that mTBI may lead to increased cortical activation in the early post-trauma period, we hypothesize that aIC activation within 2 weeks post-MVC may be elevated among probable PTSD survivors who sustained an mTBI compared to those who did not sustain mTBI. **Methods**: In this secondary hypothesis-driven analysis, previously reported task-related activation at both 2 weeks and 3 months after trauma was extracted from the aIC region of interest and compared among groups of survivors diagnosed with probable PTSD at 3 months with (*n* = 5) and without (*n* = 11) mTBI in the emergency department. An exploratory analysis of change over time included two time points and additional groups of survivors without probable PTSD with (*n* = 10) and without (*n* = 12) mTBI. **Results**: At 2 weeks, aIC activation was significantly greater in the probable PTSD with mTBI group than in the probable PTSD without mTBI group (mean = 0.125, SD = 0.053 vs. 0.014, SD = 0.111) (Welch’s *t* (13.86) = 2.71, two-sided *p* = 0.017). aIC activation in probable PTSD survivors with and without mTBI was not significantly different at 3 months after MVC. The time and time × group interactions were not significant in brain activation and post-traumatic stress symptoms, but within-group tests suggest that decreases in post-traumatic stress symptoms over time were significant in groups without mTBI. **Conclusions**: These preliminary results are consistent with the hypothesized association between mTBI and greater aIC activation to negative emotional stimuli in the early post-trauma period among survivors with probable PTSD, but do not support post-trauma brain changes in survivors with mTBI, probable PTSD, both conditions, or neither condition. These preliminary findings warrant further investigation into the relationship between mTBI and probable PTSD-related cortical alterations during the acute post-trauma period.

## 1. Introduction

Mild traumatic brain injury (mTBI) affects millions of adults each year [1]. It is characterized by a brief impairment of consciousness and/or brief periods of post-traumatic amnesia [2]. Studies suggest that mTBI is associated with an increased risk of developing post-traumatic stress disorder (PTSD) [3,4,5]. PTSD is characterized by symptoms of negative emotion dysregulation [6] and has been linked to altered activation in brain regions involved in emotion processing, including insular and limbic regions and prefrontal emotion-regulatory regions [7,8]. An increasing number of studies suggest that mTBI may affect the brain structure and function of frontal and parietal regions, which may be associated with PTSD symptom development [9,10]. We further found that mTBI may alter parietal cortical thickness and reduce visual processing of fearful faces in parietal and orbitofrontal cortices 2 weeks after a motor vehicle collision (MVC) [11,12]. These early alterations may be associated with PTSD symptom severity at 3 months after MVC [11,12]. However, whether early mTBI affects PTSD-related brain alterations in the months after trauma remains under-studied.

We further reported, in the same MVC survivors, increased anterior insular cortex (aIC) activation associated with the cognitive appraisal of negative emotion from 2 weeks to 3 months after MVC in trauma survivors who both did and did not develop probable PTSD [11], suggesting that this brain change may be observed during the early post-trauma period. Hyperactivation in the aIC has been reported in chronic PTSD [8,13,14]. The aIC plays a key role in salience detection, emotional awareness, threat appraisal, and the integration of physical and emotional states [15], and hyperactivation in the aIC may be associated with PTSD symptoms of hypervigilance [16,17]. However, the factors that may contribute to the development of aIC hyperactivation remain unclear.

mTBI is associated with reduced insular cortex structure and altered connectivity between insular and prefrontal cortices, which may impair cognitive and emotion processing involving the insular cortex as early as 1 day post-injury [18,19,20]. Individuals with comorbid traumatic brain injury (TBI) and PTSD show altered insula-centered connectivity during emotional-face processing [21]. Additionally, mTBI with PTSD has been associated with greater neural responses during emotional appraisal than mTBI alone [22]. Recent large-scale evidence further demonstrates distinct salience-network abnormalities in comorbid chronic mTBI and PTSD [23]. Based on the above findings of early mTBI effects and greater insular activation in chronic PTSD survivors with mTBI, it is likely that mTBI effects on the insular cortex in the early days to weeks after trauma may be involved in the post-trauma development of PTSD-related hyperactivation in the aIC. Therefore, we hypothesized that the previously reported aIC hyperactivation during fearful-face appraisal among survivors with probable PTSD at 3 months after MVC was greater at 2 weeks post-trauma in those who had experienced mTBI during the MVC compared to those who had not experienced mTBI. We further explored the potential post-trauma changes over 2 weeks to 3 months in all survivors with or without either mTBI, probable PTSD, or both for potentially prolonged mTBI effects. This secondary analysis integrated previously reported aIC activation during a Shifted-attention Emotion Appraisal Task (SEAT) and mTBI status, probable PTSD status, and symptom severity [11,12].

## 2. Methods

### 2.1. Participants

This report is a secondary analysis of a previously published prospective cohort of adults aged 18–60 years who experienced an MVC and presented to the emergency department (ED) within 48 h of the MVC with minor physical injury but an elevated acute post-traumatic stress symptom score on the PTSD Checklist-Stressor Specific Version (PCL) of the Diagnostic and Statistical Manual of Mental Disorders, fourth edition, text revision (DSM-IV-TR), defined as >33. Details of recruitment, data collection, PTSD assessment, and fMRI data analysis have been reported previously [11].

### 2.2. mTBI Diagnosis

mTBI was retrospectively diagnosed according to American Congress of Rehabilitation Medicine (ACRM) criteria using ED medical records and self-reported symptoms in the ED in our previous report [11]. Participants with head impact or acceleration–deceleration during MVC were classified as the mTBI (mTBI+) group if they had loss of consciousness for less than 30 min, post-traumatic amnesia for less than 24 h, or acute neurological symptoms such as disorientation, dizziness, or headache [24]. Survivors who did not meet any ACRM criteria were classified as the non-mTBI (mTBI−) group.

### 2.3. PTSD Symptom Assessment and Diagnosis

We reported post-traumatic stress symptom assessment in a previous report [11]. Briefly, post-traumatic stress symptoms (PTSS) were assessed using the PCL at both 2 weeks (T1) and 3 months (T2) after the MVC. Survivors were grouped into probable PTSD or non-PTSD (PTSD+ or PTSD−) groups at 3 months after the MVC based on PCL responses. The PTSD+ group included survivors who met DSM-IV-TR criteria for PTSD (i.e., 1 re-experiencing, 3 avoidance/numbing, and 2 hyperarousal symptoms) or partial PTSD (i.e., 1 re-experiencing, and either 3 avoidance/numbing or 2 hyperarousal symptoms), while the PTSD− group included survivors who did not meet either criterion.

### 2.4. fMRI Task and aIC Region of Interest (ROI)

Functional magnetic resonance imaging (fMRI) data acquisition and preprocessing procedures have been described previously [11]. Briefly, during the Shifted-attention Emotion Appraisal Task (SEAT), subjects viewed a grayscale composite image depicting an emotional facial expression (angry, fearful, or neutral) superimposed on an indoor or outdoor scene. On each trial, survivors determined whether: (a) the face was male or female (Male/Female) to probe implicit emotional processing, (b) the scene was indoor or outdoor (Indoor/Outdoor) to probe attention modulation of emotion, or (c) whether they liked or disliked the face (Like/Dislike) to probe modulation of emotion by cognitive appraisal.

Data were processed and analyzed using a general linear model (GLM) via FLAME-1 (FMRIB’s Local Analysis of Mixed Effects; FSL v5.2.0) with two-sample *t*-tests, controlling for age and gender. Our initial report found significantly greater activation in the left aIC (whole brain family-wise error rate (FWE) voxel z > 2.3 (*p* < 0.01) and a cluster significance threshold of *p* < 0.05) associated with like/dislike of fearful vs. neutral faces at T2, but not T1, after MVC in survivors who did and did not develop probable PTSD at T2 [11].

**Figure 1:**
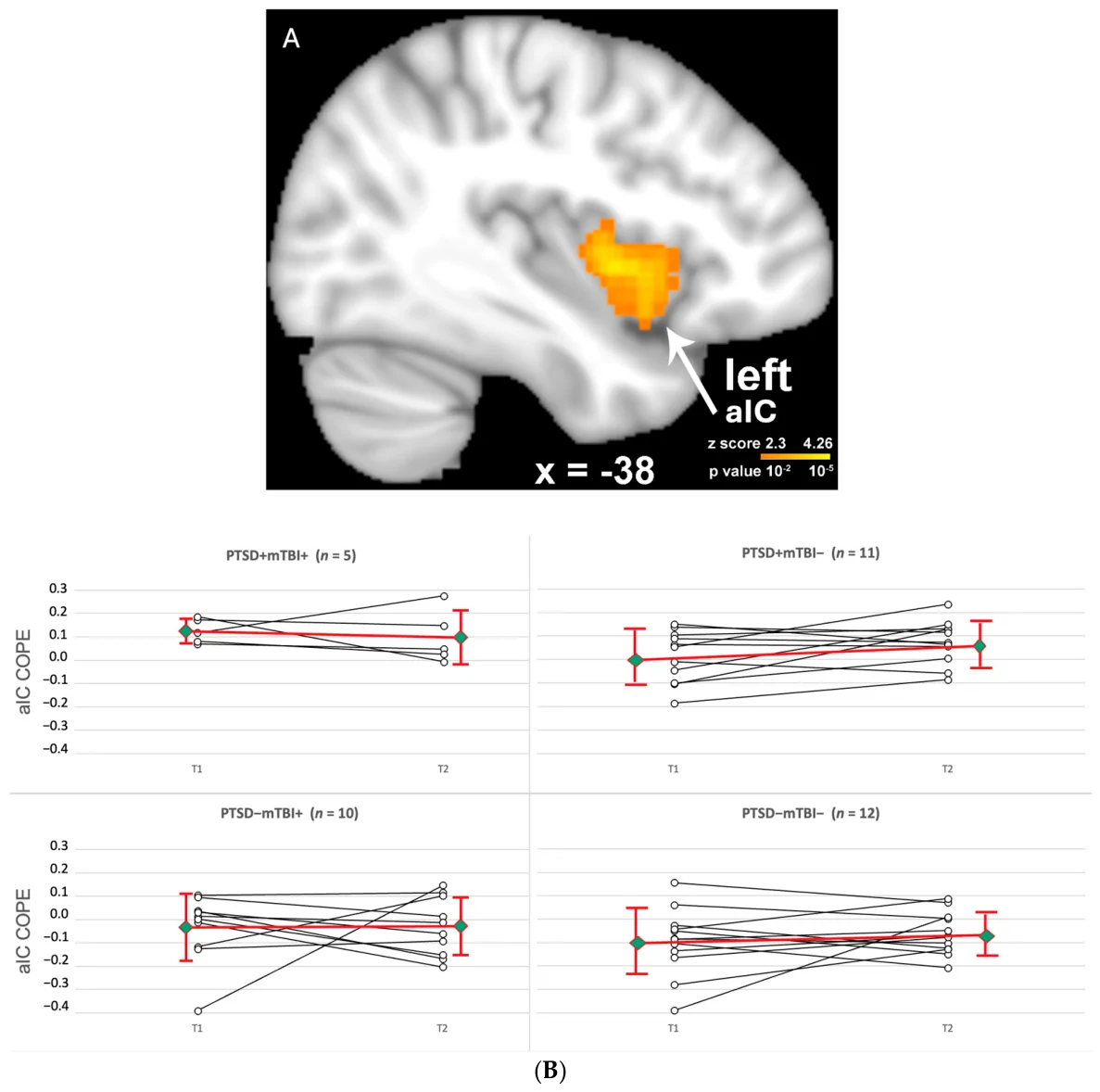
(**A**) Previously reported greater aIC activation associated with appraisal (Like/Dislike) of fearful vs. neutral faces in trauma survivors who did or did not have probable PTSD 3 months after MVC. The figure is modified from the previous report [11]. (**B**) Task-related activation (COPE) within this significant left aIC cluster was extracted at T1 and T2 after the MVC for all participants. The x-axis represents time post-trauma. Open circles connected by black lines represent individual participants. Participants were stratified into four groups: PTSD + mTBI+, PTSD + mTBI−, PTSD − mTBI+, and PTSD − mTBI−. Green diamond shapes and connecting red lines indicate group means, and red error bars represent ±1 standard deviation.

In the current secondary ROI analysis, the average percent change in contrast of parameter estimates (COPEs) was extracted from both time points using a mask derived from the clusters of left aIC voxels (−38, 2, 0, z = 3.91, cluster extent = 990 voxels; Figure 1A) with significant group difference at three months after MVC using FSL/featquery.

### 2.5. Statistical Analysis

A literature-informed, hypothesis-driven secondary analysis compared the aIC activation in PTSD + mTBI+ and PTSD + mTBI− groups at 2 weeks after an MVC using independent-samples *t*-tests; the directional hypothesis was evaluated using both one-sided and two-sided tests. A complementary *t*-test was conducted at 3 months to test potential prolonged mTBI effects. Exploratory repeated-measures general linear models (RM-GLMs) for both aIC activation and PCL scores, including all four PTSD/mTBI groups, were then used to evaluate time and group × time effects and adjusted for potential confounders of age, sex, and follow-up duration. Estimated marginal means were compared with Sidak correction, and simple effects of time were examined within each group. Age and post-trauma scan durations were compared among groups using analysis of variance (ANOVA). Statistical significance was set at *p* < 0.05.

## 3. Results

The current analysis included 38 survivors from the initial report [11]. They were divided into four groups based on probable PTSD status at T2 after MVC and mTBI status in the ED: PTSD + mTBI+ (*n* = 5), PTSD + mTBI− (*n* = 11), PTSD − mTBI+ (*n* = 10), and PTSD − mTBI− (*n* = 12) groups. No significant differences in age or post-trauma duration of the T1 scans were observed, and all groups included subjects of both sexes. The post-trauma duration of T2 scans differed among groups (F = 3.2, *p* = 0.035), with scans in the PTSD − mTBI+ group occurring significantly later than those in the PTSD − mTBI− group (*p* < 0.05) (Table 1).

The hypothesis-driven secondary analysis tested whether aIC activation during fearful-face appraisal was greater in the PTSD + mTBI+ than PTSD + mTBI− group at 2 weeks after the MVC. At T1, the results indicated that aIC activation was significantly greater in the PTSD + mTBI+ group (mean = 0.125, SD = 0.053, *n* = 5) than in the PTSD + mTBI− group (mean = 0.014, SD = 0.111, *n* = 11) (Figure 1B). Levene’s test approached significance at a trend level (*p* = 0.055) in unequal group sizes (5 vs. 11); therefore, Welch’s test was used with *t*(13.86) = 2.71, two-sided *p* = 0.017, at T1. We further explored the same group difference in this aIC activation at T2, which was not significant (means = 0.098, SD = 0.115, and 0.074, SD = 0.094, respectively); Levene’s test *p* = 0.519, and standard Student’s *t*(14) = 0.45, *p* = 0.663.

We further examined changes over 2 weeks to 3 months after MVC using time and time × group interaction in an exploratory RM-GLM including all four PTSD/mTBI groups. aIC activation differed significantly among the four groups overall, F(3, 28) = 5.48, *p* = 0.004, partial η^2^ = 0.370, whereas the main effect of time and the time × group interaction were not significant. The group effect was significant at T2, F(3, 28) = 3.55, *p* = 0.027, partial η^2^ = 0.275, but not at T1, F(3, 28) = 2.90, *p* = 0.053, partial η^2^ = 0.237; however, no individual pairwise comparison remained significant after Sidak correction at any time point.

In the exploratory repeated-measures ANOVA analysis of PCL scores, adjusted for age, sex, and follow-up duration, PCL scores differed significantly among the four PTSD/mTBI groups, F(3, 27) = 32.14, *p* < 0.001, partial η^2^ = 0.781 (*n* = 37; one participant with missing T1 PCL data was excluded) (Table 1). The overall main effect of time and the time × group interaction were not significant; F(1, 27) = 0.37, *p* = 0.549, partial η^2^ = 0.013, and F(3, 27) = 0.52, *p* = 0.671, partial η^2^ = 0.055, respectively. However, simple-effects analyses within groups showed significant decreases in PCL scores over time in the PTSD − mTBI− group (mean decrease = 10.73), Wilks’ Λ = 0.784, F(1, 27) = 7.46, *p* = 0.011, partial η^2^ = 0.216, and in the PTSD + mTBI− group (mean decrease = 9.39), Wilks’ Λ = 0.779, F(1, 27) = 7.64, *p* = 0.010, partial η^2^ = 0.221. The group effect was significant at both T1, F(3, 27) = 19.39, *p* < 0.001, partial η^2^ = 0.683, and T2, F(3, 27) = 26.49, *p* < 0.001, partial η^2^ = 0.746. Sidak-corrected pairwise comparisons showed that at T1, PCL scores were significantly higher in the PTSD + mTBI− group than in both PTSD− groups (both *p* < 0.001), while the PTSD + mTBI+ group had significantly higher PCL scores than the PTSD − mTBI+ group (*p* = 0.027). At T2, PCL scores were significantly higher in both PTSD+ groups than in both PTSD− groups (all *p* ≤ 0.008). PCL scores did not differ significantly between the PTSD + mTBI− and PTSD + mTBI+ groups at either time point.

## 4. Discussion

Mild traumatic brain injury may be associated with increased risk of developing PTSD, but the pathophysiology is not clear. This exploratory secondary analysis found that the previously reported aIC hyperactivation in probable PTSD survivors at 3 months after MVC was greater within 2 weeks after trauma among probable PTSD survivors who experienced mTBI than in those who did not experience mTBI during the MVC. These results suggest an early association between mTBI and greater aIC emotion-related activation among survivors with probable PTSD. However, the complementary analyses did not indicate that mTBI significantly influenced changes in aIC activation over time and at 3 months after MVC. Interestingly, post-traumatic stress symptoms improved significantly over time in survivors without mTBI, but not significantly in those with mTBI. This pattern is consistent with improvement in post-traumatic stress symptoms in MVC survivors who did not experience mTBI. However, the nonsignificant time and time × group interactions do not support significant effects of mTBI on symptom trajectories.

Studying the mechanisms of early mTBI effects on PTSD-related insular changes is beyond the scope of this study; however, several speculative mechanisms may contribute to the early functional changes observed after mTBI. We previously reported mTBI-related early cortical thinning and reduced activation associated with viewing fearful faces in the left superior parietal cortex at 2 weeks after MVC in these subjects [12]. Alterations in visual processing of fearful emotional information may alter sensory inputs to the aIC through the dorsal stream of visual processing [25,26]. On the other hand, we also reported reduced orbitofrontal cortical activation during viewing of fearful faces in the same subjects with mTBI compared to those without mTBI at 2 weeks after trauma [12]. The orbitofrontal gyrus (OFG) is involved in the top-down inhibitory regulation of negative emotion processing from the prefrontal cortex (PFC) to the aIC and limbic regions [27]. Reduced activation in this emotion-regulation process could theoretically contribute to greater emotion-related activation in the aIC after mTBI. Finally, acute mTBI has also been associated with abnormal coupling between the aIC and brain regions in the salience network or default mode network, which may reflect disruption of large-scale network switching and salience processing [19,20,21]. MVC survivors who later developed PTSD exhibited more white matter abnormalities in the acute phase than non-PTSD survivors [28]. Therefore, mTBI may be associated with reduced connectivity between the insula and the PFC during the early post-trauma period [20]. Our preliminary findings highlight the need for future systematic investigations into mTBI effects on the aIC and their possible role in the development of PTSD.

The current findings suggest that mTBI may be associated with greater aIC activation early after trauma in probable PTSD survivors. However, this influence was not distinguishable after 2 weeks and at 3 months post-trauma. The causes of this acute, but potentially transient, difference remain unclear, and the findings should be interpreted cautiously given the limited data. We previously reported mTBI-related cortical thickening in the left rostral middle frontal gyrus region at 2 weeks after MVC, followed by further decreases over the subsequent 3 months [29], suggesting that brain changes after mTBI may evolve. Whether these mTBI-related changes only transiently influence probable PTSD-related brain alterations warrants further investigation. However, in our previous report, we also reported decreases in left dorsomedial PFC structure and activation during the appraisal of fearful faces from 2 weeks to 3 months after MVC in survivors who exhibited probable PTSD [11]. We speculated that the observed aIC hyperactivation at 3 months after MVC may reflect reduced prefrontal inhibitory control [11]. Reduced medial PFC inhibition over time could theoretically affect both mTBI and non-mTBI survivors who are developing probable PTSD, which could partially explain the nonsignificant difference in aIC hyperactivation between probable PTSD groups with and without mTBI at 3 months after MVC. However, this interpretation remains speculative. If plausible, it may align with a multidimensional view of the pathogenesis of PTSD. Therefore, future longitudinal studies are clearly needed to explore the possible influence of mTBI and other factors on PTSD-related brain changes after trauma.

## 5. Limitations

The current preliminary study has several limitations. The present results are based on limited sample sizes and the classification of probable PTSD based on self-reports. A single trauma type of MVC limits generalizability of findings to other types of traumas. The findings across two time points do not provide evidence for causality, and the nonsignificant time and time × group interactions require further examination of post-trauma changes in brain activation. The hypothesis-driven *t*-test of group comparison between probable PTSD survivors with and without mTBI at T1 was selected for limited group sizes and independent of probable PTSD-related ROI selection. However, the RM-GLM findings at T2 are not an independent replication of the previously reported probable PTSD effect. The present RM-GLM results within the aIC ROI should therefore be considered exploratory.

## 6. Conclusions

This exploratory study on previously reported hyperactivation in the left aIC to negative emotional stimuli in survivors with probable PTSD found greater aIC activation during the early post-trauma period, within 2 weeks, among probable PTSD survivors with mTBI compared to those without mTBI. These findings suggest a potential early post-trauma association between mTBI and brain alterations related to probable PTSD. However, further exploratory tests in the subsequent 3 months did not support post-trauma change in probable PTSD symptoms or additional mTBI influences, except a decrease in PTSD symptom severity in survivors without mTBI in a within-group comparison of two time points. The preliminary findings support future studies on the potential influence of mTBI on brain alterations in survivors with probable PTSD.

## Figures and Tables

**Table 1:** Sample characteristics.

Group	PTSD − mTBI−	PTSD − mTBI+	PTSD + mTBI−	PTSD + mTBI+
N	12	10	11	5
Sex, male/female	3/9	3/7	4/7	2/3
Age, years	35.25 ± 14.07	34.00 ± 12.87	32.45 ± 10.55	29.60 ± 7.50
Time 1 scan post-trauma days	9.58 ± 4.87	7.90 ± 3.35	12.55 ± 4.48	7.80 ± 4.60
Time 2 scan post-trauma days	100.58 ± 13.52	118.50 ± 14.6	108.64 ± 13.16	113.20 ± 14.25
Time 1 PCL scores	30.83 ± 6.19	31.40 ± 11.51	60.55 ± 10.53	48.00 ± 15.64
Time 2 PCL scores	22.42 ± 5.84	26.00 ± 9.70	52.36 ± 10.00	44.20 ± 10.80
Time 1 aIC activation (COPE)	−0.10 ± 0.14	−0.04 ± 0.15	0.01 ± 0.11	0.13 ± 0.05
Time 2 aIC activation (COPE)	−0.06 ± 0.09	−0.03 ± 0.13	0.07 ± 0.09	0.10 ± 0.11

## Data Availability

Data are available upon request from Xin Wang at the Departmental Research Committee of the Department of Psychiatry at the University of Toledo for researchers who meet the criteria for access to confidential data.

## Abbreviations

The following abbreviations are used in this manuscript:

mTBI	Mild traumatic brain injury
PTSD	Post-traumatic stress disorder
aIC	Anterior insular cortex
MVC	Motor vehicle collision
SEAT	Shifted-attention Emotion Appraisal Task
PCL	PTSD Checklist-Stressor Specific Version
DSM-IV-TR	Diagnostic and Statistical Manual of Mental Disorders, Fourth Edition, Text Revision
PTSSs	Post-traumatic stress symptoms
fMRI	Functional magnetic resonance imaging
ACRM	American Congress of Rehabilitation Medicine
T1	2 weeks post-trauma
T2	3 months post-trauma
GLM	General linear model
FLAME-1	FMRIB’s Local Analysis of Mixed Effects
FSL	FMRIB Software Library
RM-GLM	Repeated-measures general linear model
ANOVA	Analysis of variance
ROI	Region of interest
FWE	Family-wise error rate
COPE	Contrast of parameter estimates
OFG	Orbitofrontal gyrus
PFC	Prefrontal cortex
